# Supplementary material for: Unveiling the nature of Pt-induced anti-deactivation of Ru for alkaline hydrogen oxidation reaction
Source: Nat Commun. 2024 Feb 22;15:1614. doi: 10.1038/s41467-024-45873-0 (PMC10884033; doi:10.1038/s41467-024-45873-0)
Supplement: Supplementary file 1 — Supplementary Information [file 41467_2024_45873_MOESM1_ESM.pdf]

Unveiling the Nature of Pt-induced Anti-deactivation of Ru for Alkaline Hydrogen Oxidation Reaction

Yanyan Fang[1,#], Cong Wei[1,#], Zenan Bian[1], Xuanwei Yin[1], Bo Liu[1], Zhaohui Liu[1], Peng Chi[1], Junxin Xiao[1], Wanjie Song,[1] Shuwen Niu[1], Chongyang Tang[1], Jun Liu[2], Xiaolin Ge,[1] Tongwen Xu,[1] Gongming Wang[1,*]

[1] Department of Chemistry, University of Science and Technology of China, Hefei 230026, China

[2] Institute of Solid State Physics, Hefei Institutes of Physical Science, Chinese Academy of Sciences, Hefei 230031, China.

[#] These authors contributed equally: Yanyan Fang and Cong Wei

[*] Corresponding author. E-mail: wanggm@ustc.edu.cn

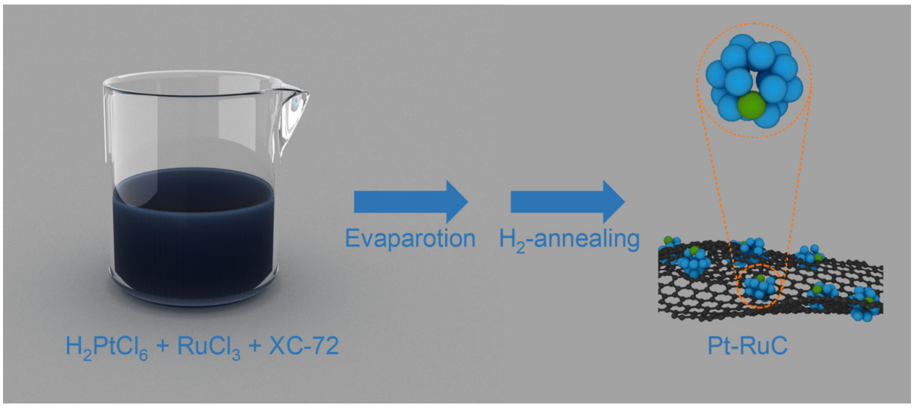

Supplementary Figure 1. Synthesis process of Pt-Ru/C. The aqueous mixture containing $RuCl_3$, H_2PtCl_6 and carbon black (XC-72) was evaporated to collect the powder for post annealing in H_2 atmosphere to obtain the Pt-Ru/C catalyst.

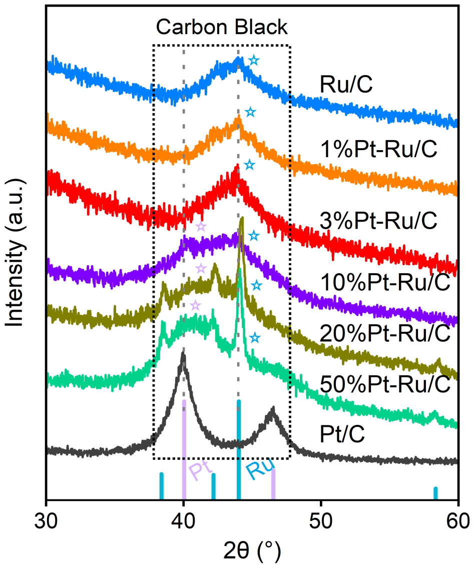

Supplementary Figure 2. XRD of Ru/C, 1%Pt-Ru/C, 3%Pt-Ru/C, 10%Pt-Ru/C, 20%Pt-Ru/C, 50%Pt-Ru/C and Pt/C. The Ru mass loading of Pt-Ru/C samples is ~ 10 wt% vs. carbon black, while the Pt mass loading of Pt/C is ~ 20 wt% vs. carbon black. The percentages of Pt in Pt-Ru/C, such as 3%Pt-Ru/C, are the atomic ratios of Pt to Ru. The wide peak labeled by the black dash framework is attributed to the diffraction peak of carbon black.

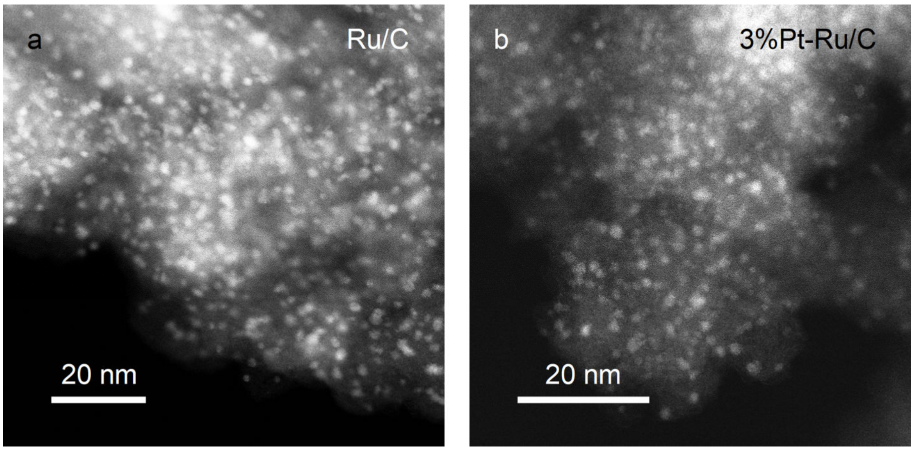

Supplementary Figure 3. HAADF-STEM images of Ru/C (**a**) and 3%Pt-Ru/C (**b**) with low magnification. Both Ru/C and 3%Pt-Ru/C show uniform distribution of metal nanoparticles on the carbon black support.

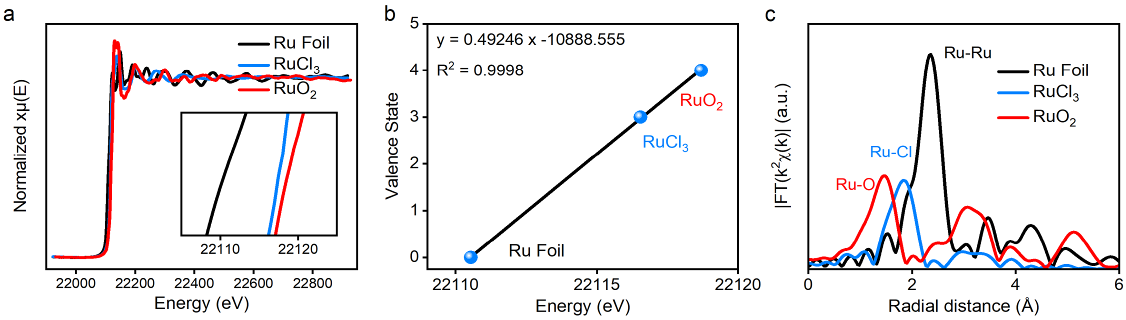

Supplementary Figure 4. a, Ru-K edge XAS of the standard materials (Ru foil, $RuCl_3$ and RuO_2). The inset shows the absorption edges. **b,** The linear fitting of valence state vs. absorption edge. **c,** The corresponding FT-EXAFS of Ru foil, $RuCl_3$ and RuO_2.

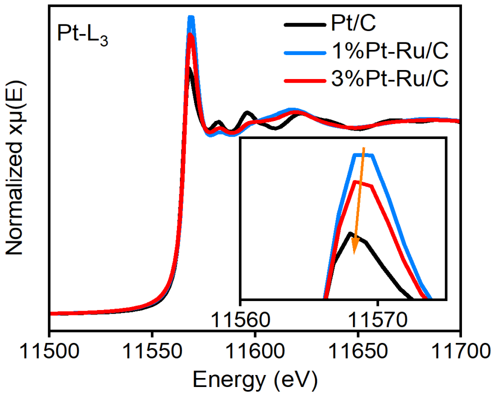

Supplementary Figure 5. Pt-L_3 edge XANES of Pt/C and Pt-Ru/C with different Pt loadings. The white line peak intensity is decreased with the increased Pt loading, indicating the gradually decreased Pt valence state.

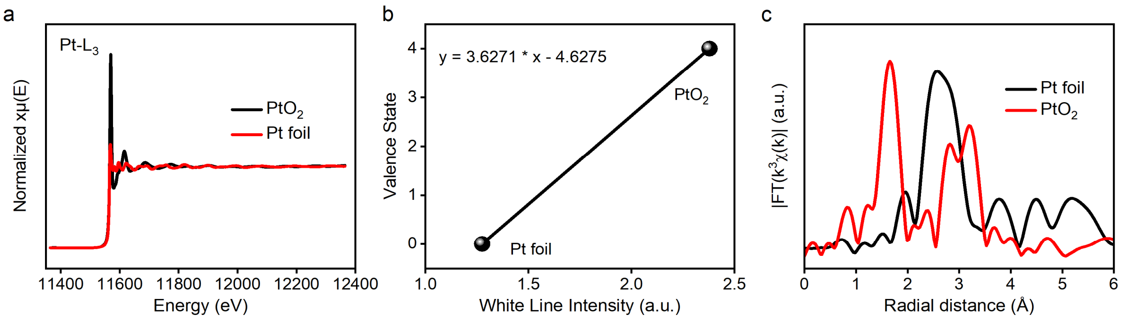

Supplementary Figure 6. a, Pt-L_3 edge XAS of the standard materials (Pt foil and PtO_2). **b,** The linear fitting of valence state vs. white line peak intensity. **c,** The corresponding FT-EXAFS.

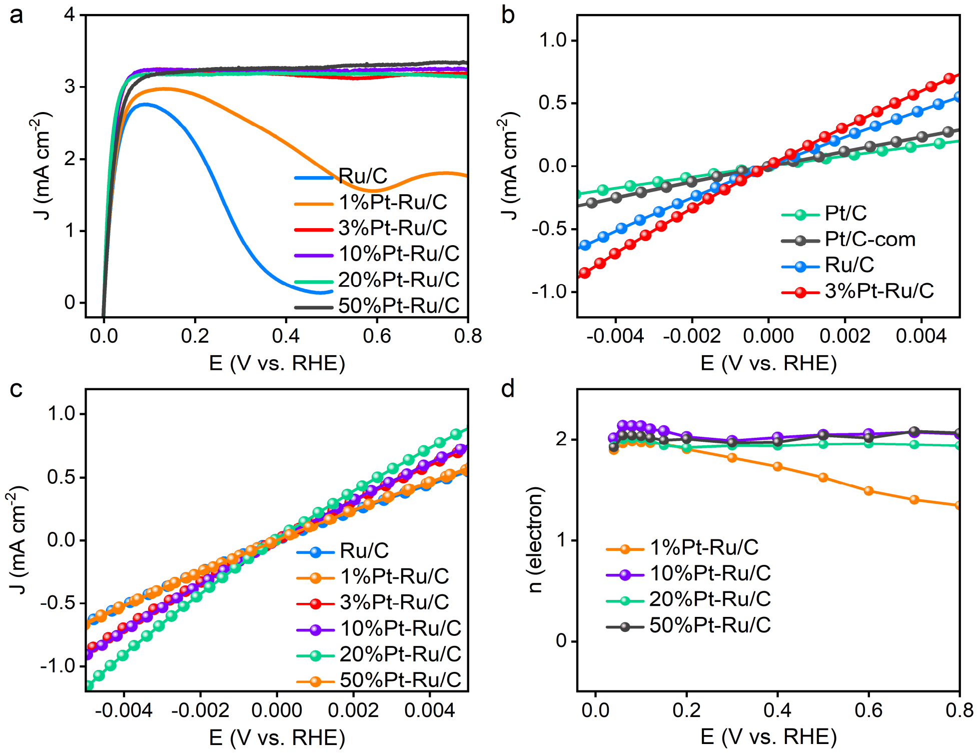

Supplementary Figure 7. HOR performance of the studied materials. **a,** Polarization curves of Ru/C, 1%Pt-, 3%Pt-, 10%Pt-, 20%Pt- and 50%Pt-Ru/C. **b, c,** Micro-polarization regions (-5 mV to 5 mV) of the studied materials. **d,** Calculated number of electrons involved in the HOR catalysis based on Koutecky–Levich equation. Compared with Ru/C, after Pt modification, not only the exchange current density of the catalyst is enhanced, but also the stable current at high anodic potential is obtained and the 2-electron HOR process is almost preserved.

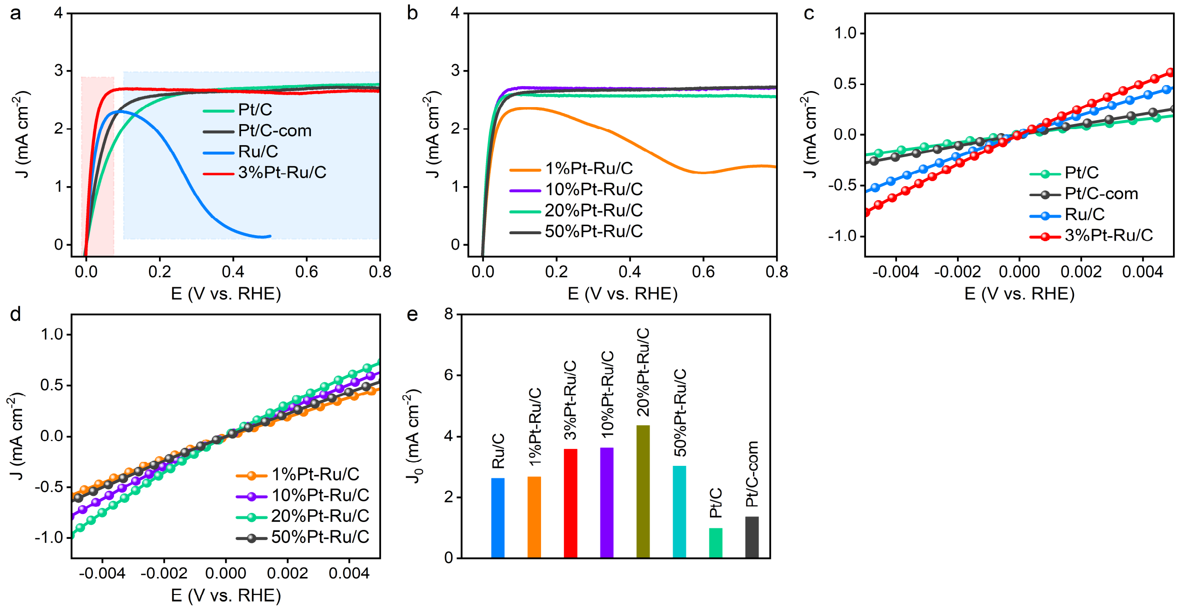

Supplementary Figure 8. HOR performance test at 1600 rpm of the studied materials. **a, b,** Polarization curves. **c, d,** Micro-polarization regions (-5 mV to 5 mV). **e,** Calculated exchange current densities. The polarization curves collected at 1600 rpm show the same trend with those collected at 2500 rpm. In brief, the catalyst deactivation at high potential is suppressed with the introduction of Pt. Meanwhile, the Pt incorporation could further enhance the exchange current density of the catalyst before the Pt atomic ratio reaches 50%.

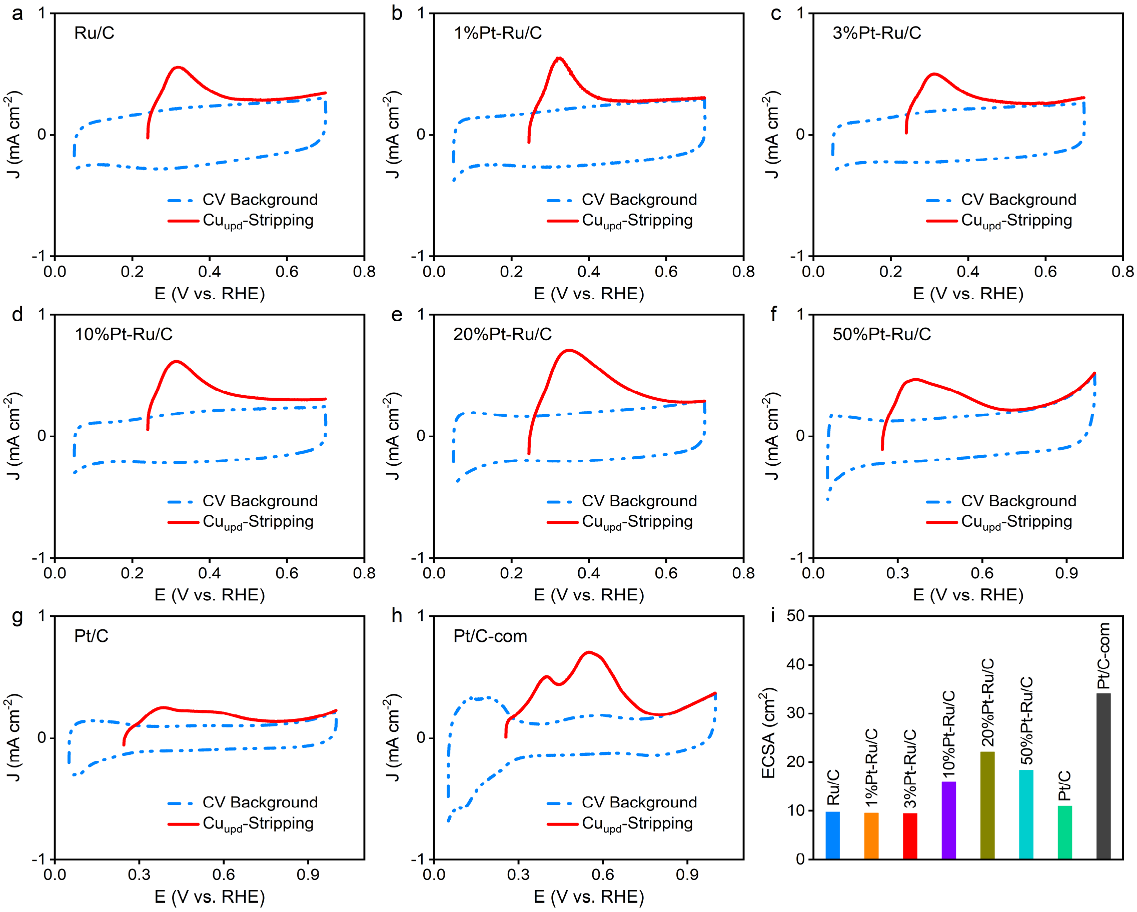

Supplementary Figure 9. a-h, The Cu_{upd}-stripping test in Ar-saturated 0.5 M H_2SO_4 of the studied materials. **i,** The calculated ECSA. With the increased Pt doping amount, the ECSA gradually increases and reaches to the highest value for 20%Pt-Ru/C, followed by ECSA loss with higher Pt loading.

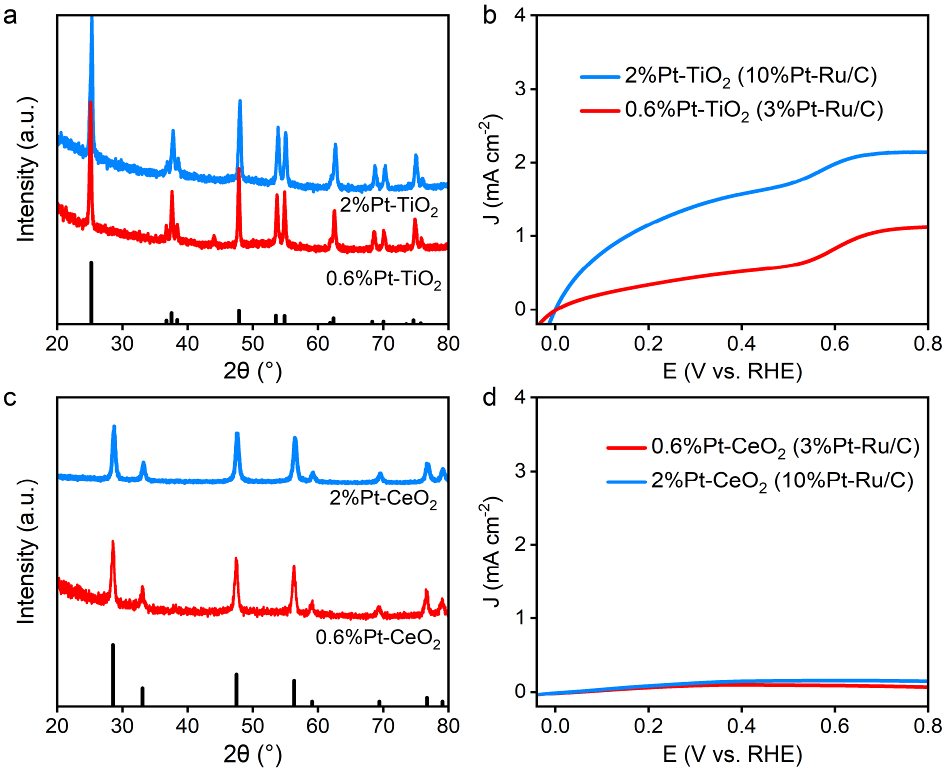

Supplementary Figure 10. HOR performance of Pt decorated CeO_2 and TiO_2. XRD patterns of Pt-TiO_2 (**a**) and Pt-CeO_2 (**b**). HOR polarization curves of Pt-TiO_2 (**c**) and Pt-CeO_2 (**d**). The Pt mass loadings on these metal oxides are controlled to be the same as those on Ru/C. In detail, 3%Pt- and 10%Pt-Ru/C contain 0.6 wt% and 2 wt% Pt, respectively. Thus, Pt with the same mass loadings were introduced onto these metal oxides. Based on the XRD patterns, no obvious signal of Pt species is observed, excluding Pt segregation. In addition, the HOR performance of Pt loaded TiO_2 and CeO_2 are far poorer than Pt-Ru/C, indicating Pt itself with low mass loading might not be efficient enough to catalyze HOR.

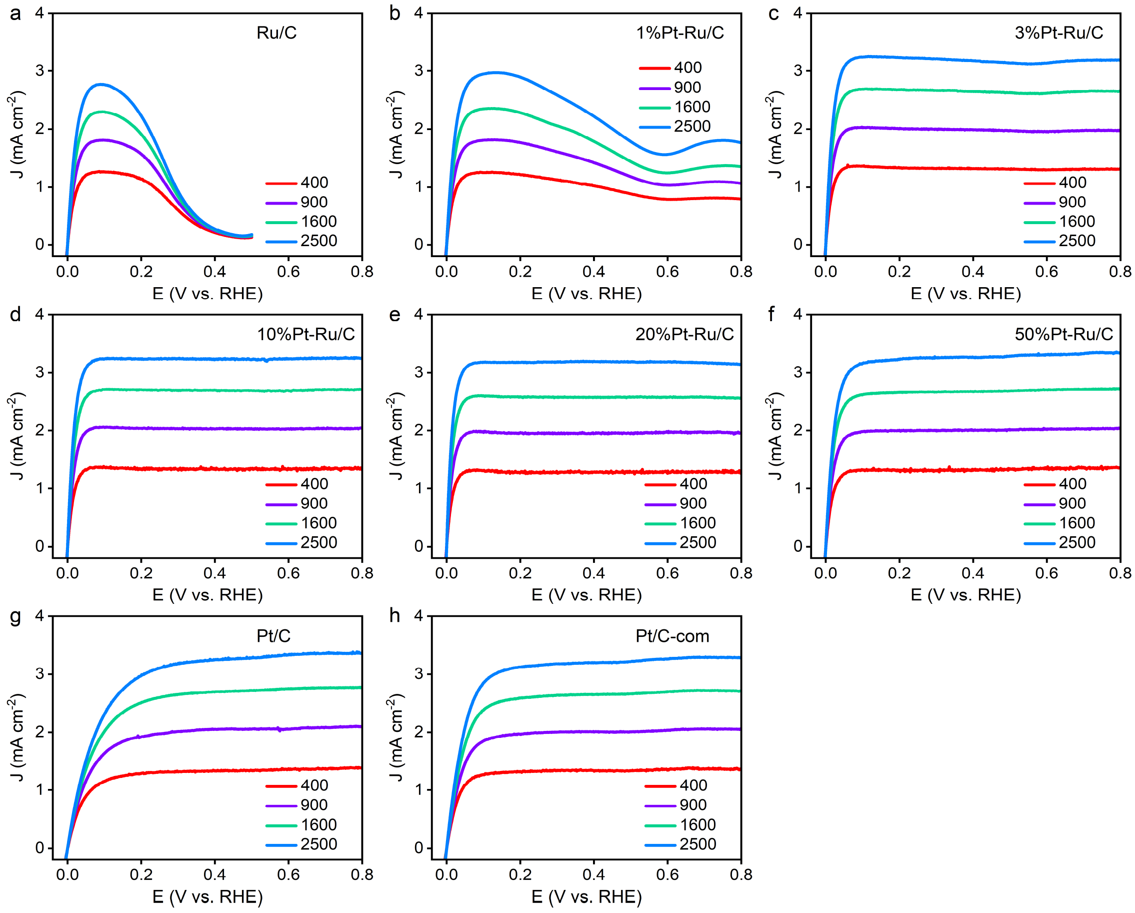

Supplementary Figure 11. LSV curves of Ru/C (**a**), 1%Pt-Ru/C (**b**), 3%Pt-Ru/C (**c**), 10%Pt-Ru/C (**d**), 20%Pt-Ru/C (**e**), 50%Pt-Ru/C (**f**), Pt/C (**g**) and Pt/C-com (**h**) obtained at different rotating speeds (400, 900, 1600 and 2500 rpm).

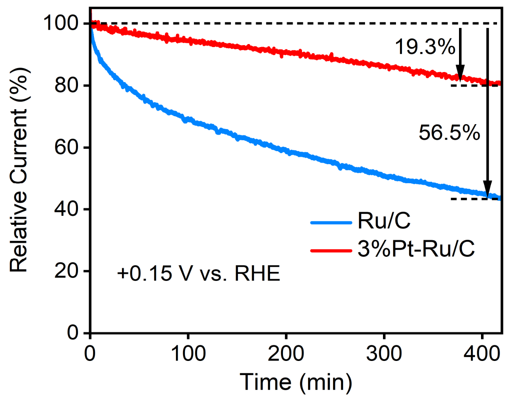

Supplementary Figure 12. The stability tests of Ru/C and 3%Pt-Ru/C using RDE method. After around 400 min chronoamperometry test, the relative current density of Ru/C shows a huge decrease of 56.5%, while the decrease of 3%Pt-Ru/C (19.3%) is much less significant, demonstrating better stability of 3%Pt-Ru/C towards HOR catalysis.

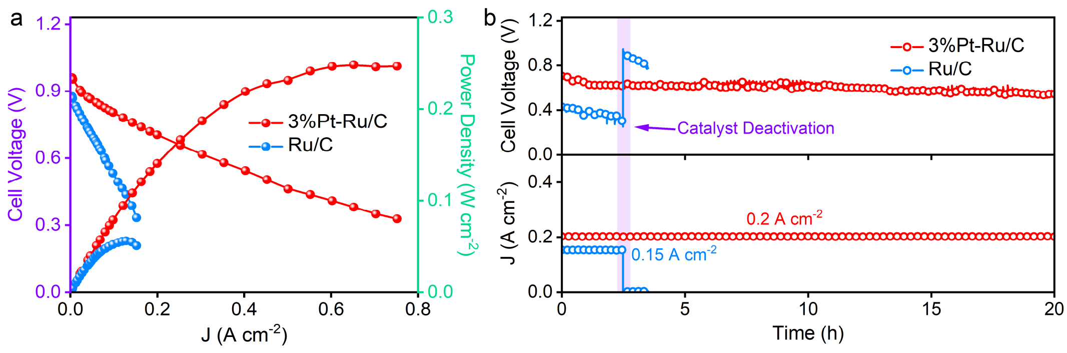

Supplementary Figure 13. a, H_2/O_2 AEMFC polarization plots with Ru/C and 3%Pt-Ru/C as the anode catalysts. **b,** Long-term stability test of the fuel cells with 3%Pt-Ru/C anode catalyst at 0.2 A cm^{-2} and Ru/C anode catalyst at 0.15 A cm^{-2}. For the cell using Ru/C anode, as current density increases, the quick cell voltage drop and limited power density demonstrate its poor performance. Besides, the cell voltage reaches to the lower limit (0.25 V) of the equipment at the current density less than 0.2 A cm^{-2}. In comparison, the power density and cell voltage using 3%Pt-Ru/C anode are both much higher, and the current density reaches to 0.75 A cm^{-2}. For the stability test, the cell with 3%Pt-Ru/C delivers stable voltage and current density output at 0.2 A cm^{-2} for at least 20 hours, while the cell with Ru/C quickly becomes deactivated even at smaller current density of 0.15 A cm^{-2}. The results of the AEMFC tests further demonstrate the much higher activity and stability of 3%Pt-Ru/C towards alkaline HOR in AEMFC.

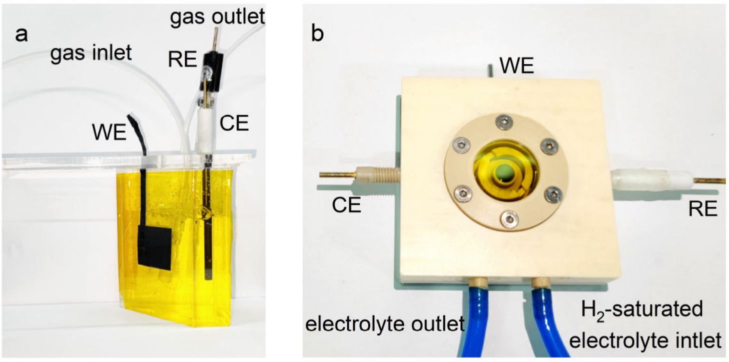

Supplementary Figure 14. Electrochemical cells for operando XAS (**a**) and SR-FTIR (**b**) tests.

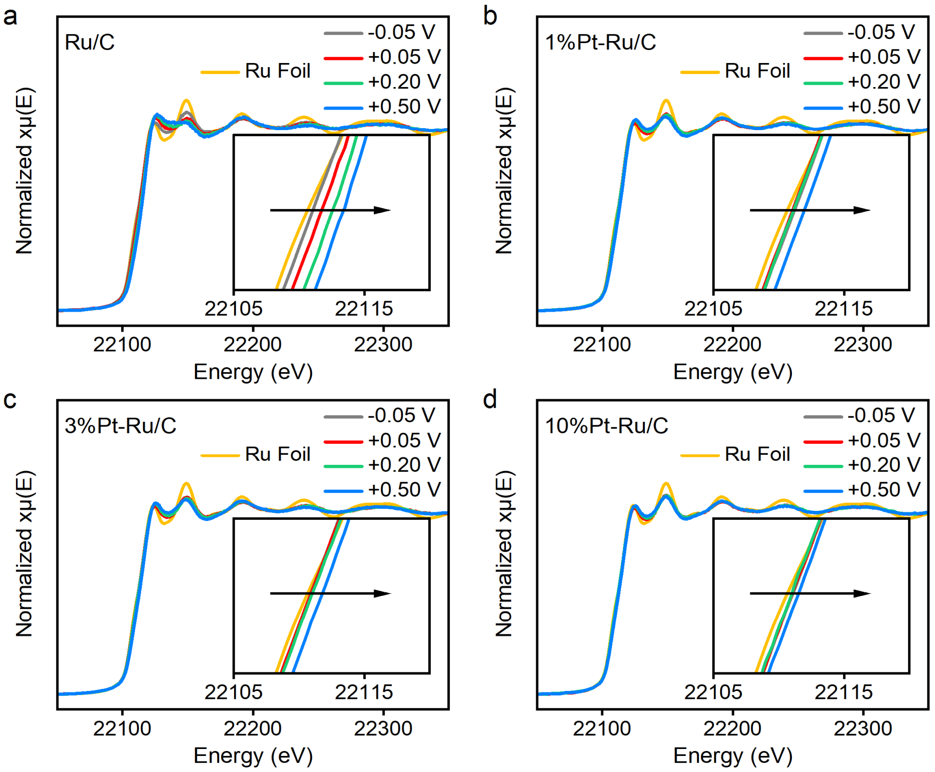

Supplementary Figure 15. Operando Ru-K edge XAS of Ru/C (**a**), 1%Pt-Ru/C (**b**), 3%Pt-Ru/C (**c**) and 10%Pt-Ru/C (**d**). The insets show the absorption edges. The whole operando Ru-K edge XAS spectra of Ru/C and different Pt-Ru/C resembles that of metallic Ru foil, indicating the metallic state of the studied materials is maintained under working conditions. However, as anodic potential increases, the absorption edge of RuC obviously shifts to higher energy, while after introducing Pt (1%, 3% and 10% atomic ratio), the blueshift is limited, proving the anti-oxidation feature after Pt doping.

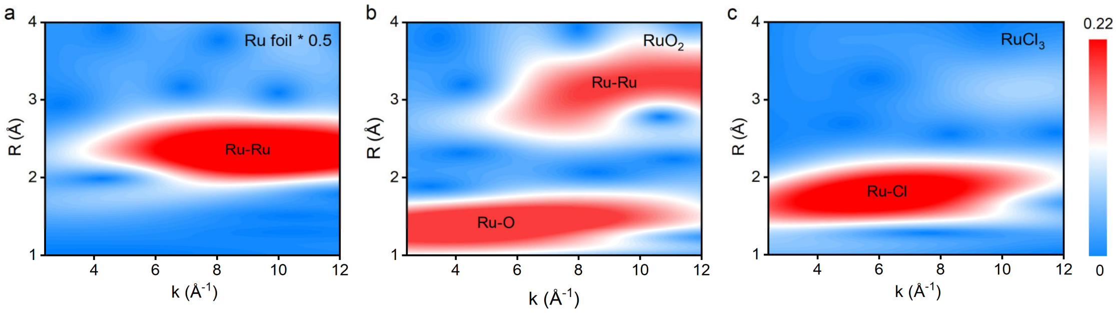

Supplementary Figure 16. Ru-K edge WT-EXAFS of Ru foil (**a**), RuO_2 (**b**) and $RuCl_3$ (**c**).

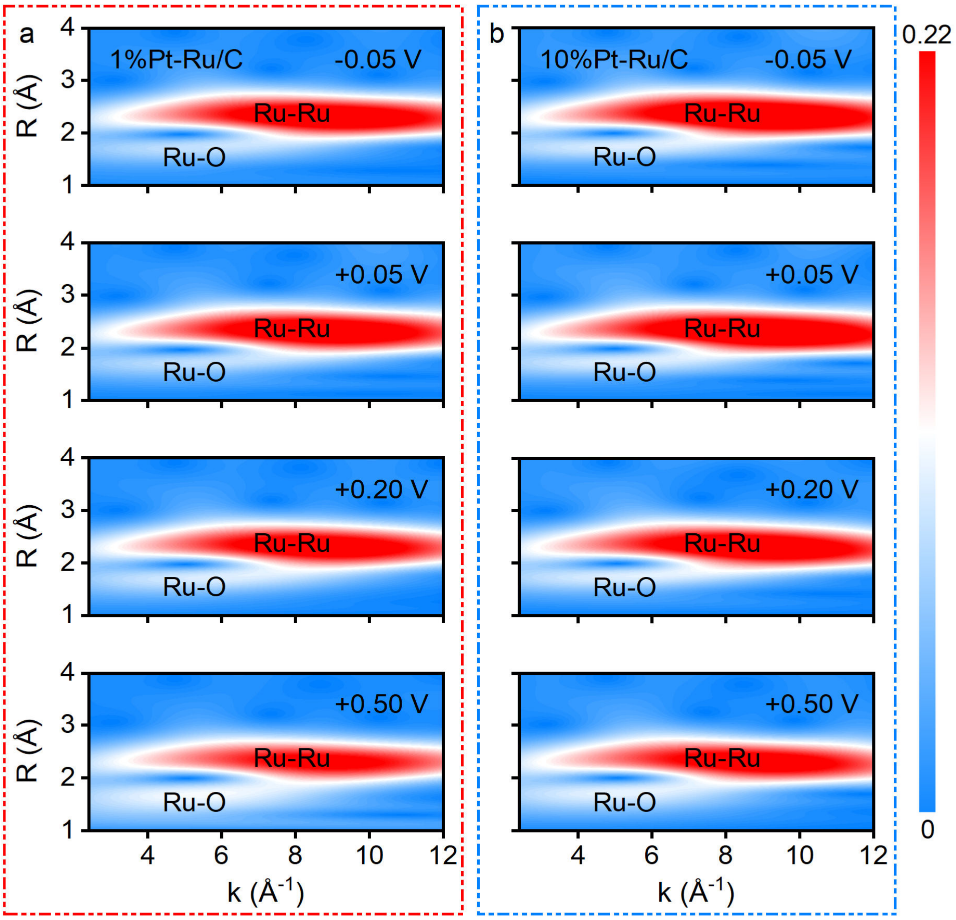

Supplementary Figure 17. Operando Ru-K edge WT-EXAFS of 1%Pt-Ru/C (**a**) and 10%Pt-Ru/C (**b**) at different potentials. With the increased potential, for both 1%Pt-Ru/C and 10%Pt-Ru/C, the increase of Ru-O is not significant and the intensity of Ru-Ru is not sensitive to high potential, indicating the robustness of the catalysts against oxidation after Pt modification.

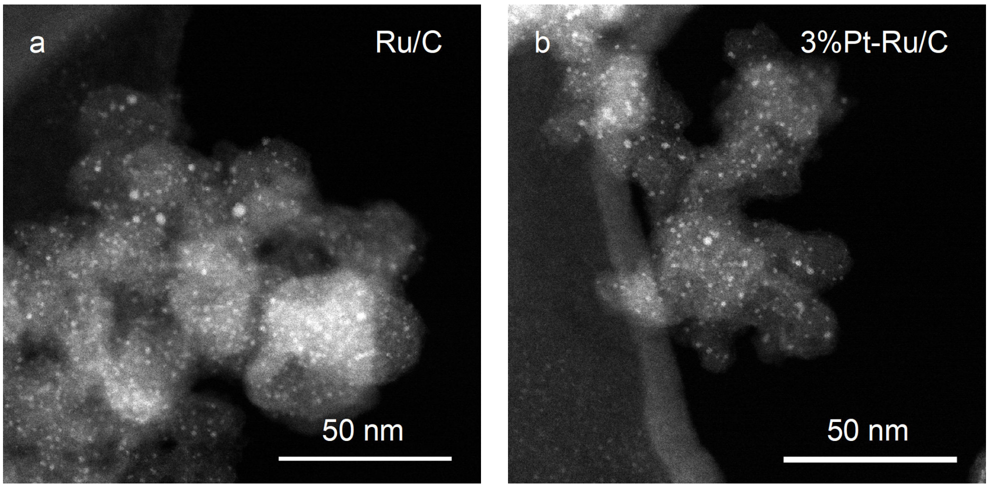

Supplementary Figure 18. HAADF-STEM images of Ru/C (**a**) and 3%Pt-Ru/C (**b**) activated at -0.05 V with low magnification. After the activation, both Ru/C and 3%Pt-Ru/C preserve homogeneous distribution of metal nanoparticles on the carbon black support.

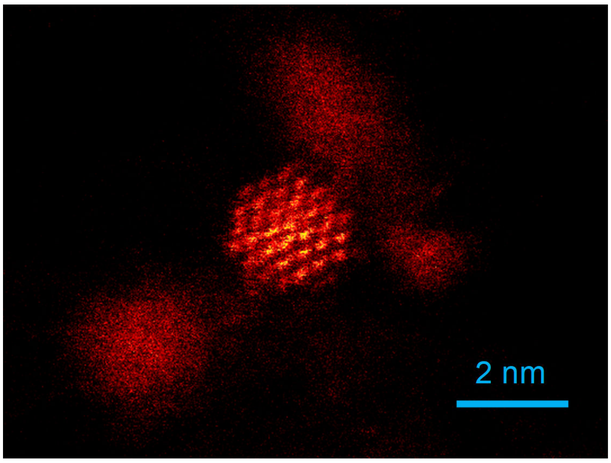

Supplementary Figure 19. HAADF-STEM image of Ru/C activated at -0.05 V. In comparison with the emergence of the bright dots at the nanoparticle edge of 3%Pt-Ru/C (Fig. 3e), the nanoparticle of Ru/C shows overall uniform brightness, demonstrating the existence of Pt single atoms on the Ru NPs of 3%Pt-Ru/C.

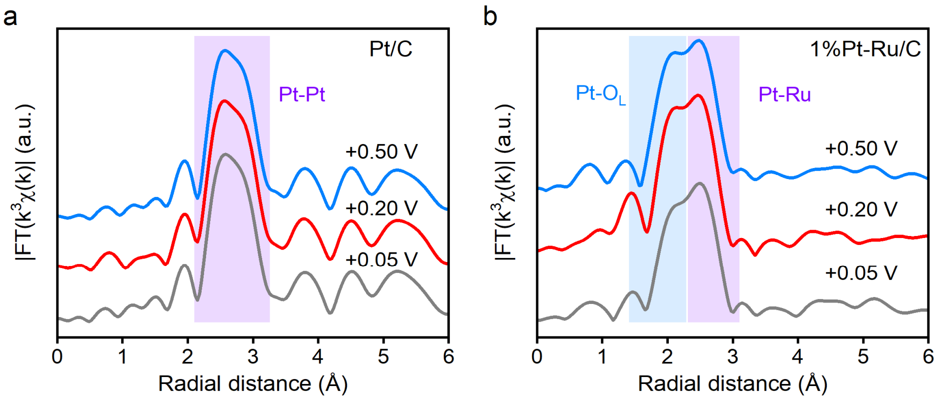

Supplementary Figure 20. Operando Pt-L_3 edge FT-EXAFS of Pt/C (**a**) and 1%Pt-Ru/C (**b**). The coordination structure of 1%Pt-Ru/C is similar to that of 3%Pt-Ru/C and different from that of Pt/C. The Pt-Ru bond in 1%Pt-Ru/C and 3%Pt-Ru/C is apparent shorter than Pt-Pt bond in Pt/C. The existence of adsorption-related Pt-O_L bond in both 1%Pt-Ru/C and 3%Pt-Ru/C indicates the exposure of Pt atoms on the particle surface. For Pt/C, the Pt-O_L is not detected because the percentage of surface Pt atoms is low and the majority of Pt atoms exist in the inner part of the nanoparticle.

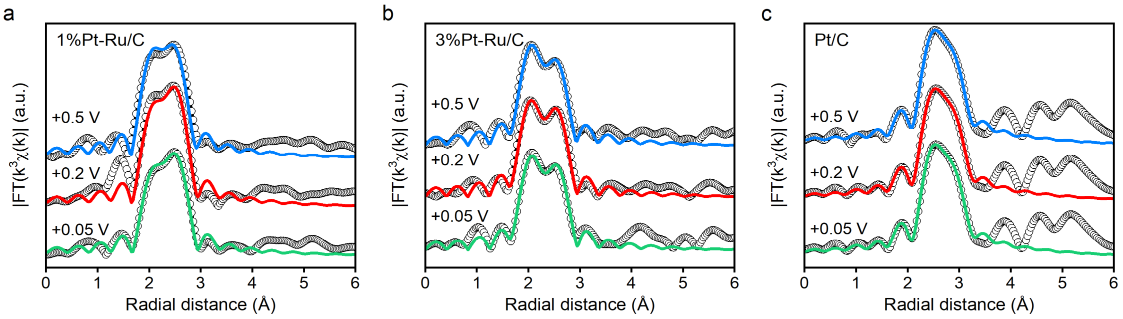

Supplementary Figure 21. Pt-L$_3$ edge EXAFS fitting of 1%Pt-Ru/C (**a**) and 3%Pt-Ru/C (**b**) using Pt-O and Pt-Ru paths, together with Pt/C (**c**) using Pt-Pt path at different potentials.

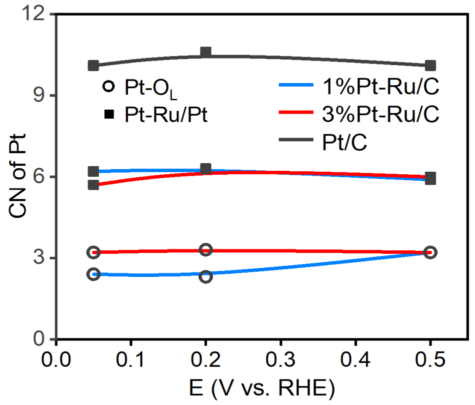

Supplementary Figure 22. The coordination numbers (CN) of Pt atom in different samples based on the Pt-L$_3$ edge EXAFS fitting results. The Pt atoms in both 1%Pt-Ru/C and 3%Pt-Ru/C are coordinated by ~3 adsorption-related O atoms and ~6 Ru atoms, which indicates the Pt single atoms share the similar chemical environment in both samples. Meanwhile, for all the samples, the coordination structure of Pt remains almost unchanged under the operando conditions, verifying the stable structure of Pt atoms during the reaction process.

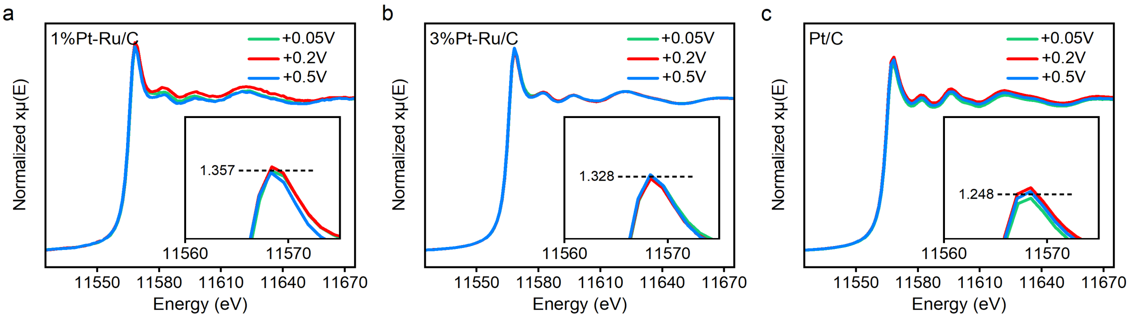

Supplementary Figure 23. Operando Pt-L_3 edge XAS of 1%Pt-Ru/C (**a**), 3%Pt-Ru/C (**b**), Pt/C (**c**). The insets show the white line peaks. For each sample, as potential increases, the white line peak intensity is not significantly changed, indicating the unchanged valence state of Pt atoms under reaction conditions. However, for the sample with higher Pt loading, the white line peak intensity is lower (from ~1.357 of 1%Pt-Ru/C to ~1.328 of 3%Pt-Ru/C and ~1.248 of Pt/C), which might originate from the overall decreased valence state of the metal nanoparticles, further suggesting the existence of Pt could protect the nanoparticle from oxidation.

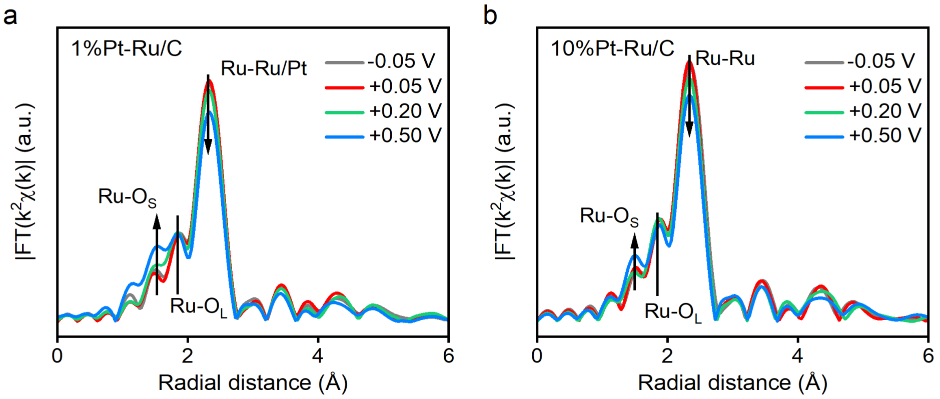

Supplementary Figure 24. Operando Ru-K edge EXAFS of 1%Pt-Ru/C (**a**) and 10%Pt-Ru/C (**b**). After introducing Pt, the increase of oxidation-related Ru-O_S is inhibited and the surface-adsorption related Ru-O_L is preserved, verifying the Pt incorporation could protect the Ru NPs from oxidation and maintain surface adsorption behavior.

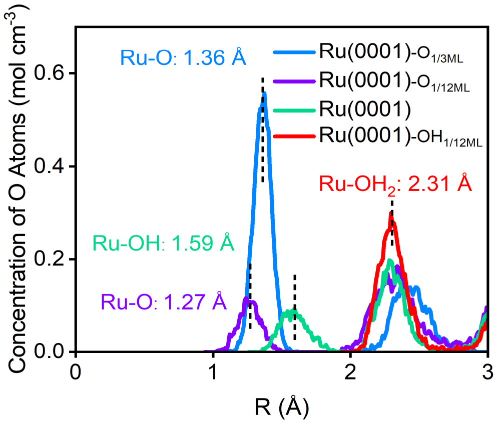

Supplementary Figure 25. Concentration distribution profiles of O atoms along the surface normal direction form the AIMD simulations. For Ru(0001)/H_2O, O atoms adsorbed on the surface only exist in the form of water molecules, which locate at ~ 2.31 Å to the surface. For Ru(0001)-$O_{1/12ML}$/H_2O and Ru(0001)-$O_{1/3ML}$/H_2O, in addition to the adsorbed water, O* atoms are also directly embedded on the surface within the range of 1.27 to 1.36 Å to the surface. For Ru(0001)-$OH_{1/12ML}$/H_2O, the adsorbed OH* species appear at 1.59 Å to the surface, which is closer to O* atoms instead of water. Thus, the Ru-O_L is mainly attributed to surface adsorbed water, while Ru-O_S contains O* and OH*. Detailed description on the AIMD simulations will be discussed later.

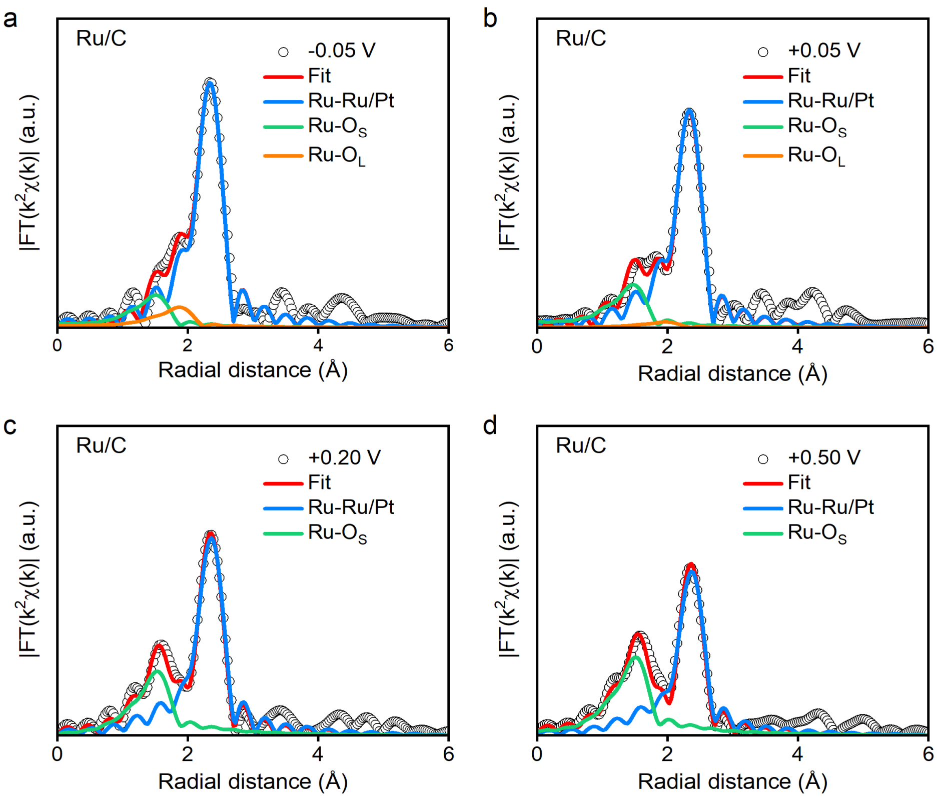

Supplementary Figure 26. Ru-K edge EXAFS fitting of Ru/C with Ru-O$_S$, Ru-O$_L$ and Ru-Ru paths at -0.05 V (**a**), +0.05 V (**b**), +0.20 V (**c**) and +0.50 V (**d**). As the potential increases, the intensity of oxidation-related Ru-O$_S$ is strengthened and the metallic feature of Ru-Ru is weakened due to surface oxidation. More importantly, the adsorption-related Ru-O$_L$ vanishes, indicating surface oxidation would lead to unfavorable surface adsorption behavior.

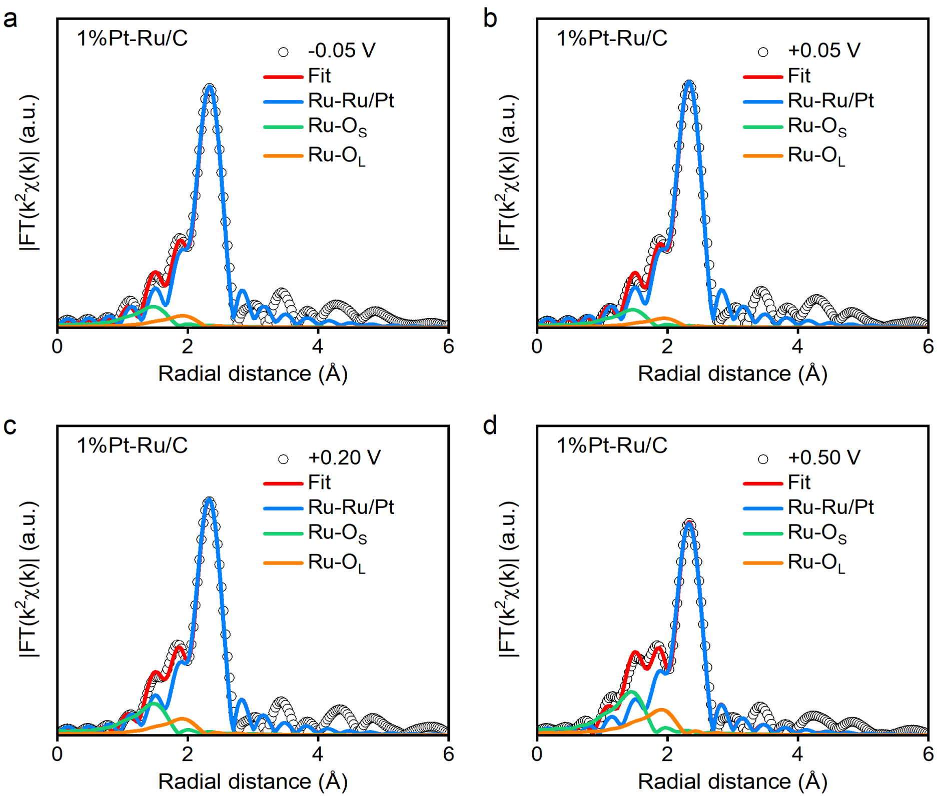

Supplementary Figure 27. Ru-K edge EXAFS fitting of 1%Pt-Ru/C with Ru-O_S, Ru-O_L and Ru-Ru paths at -0.05 V (**a**), +0.05 V (**b**), +0.20 V (**c**) and +0.50 V (**d**). As potential increases, the change of both Ru-O_S and Ru-O_L is not impressive, demonstrating the oxidation-proof surface could preserve surface water adsorption. Similar phenomenon is observed for 3%Pt-Ru/C in Supplementary Figure 21 and 10%Pt-Ru/C in Supplementary Figure 22.

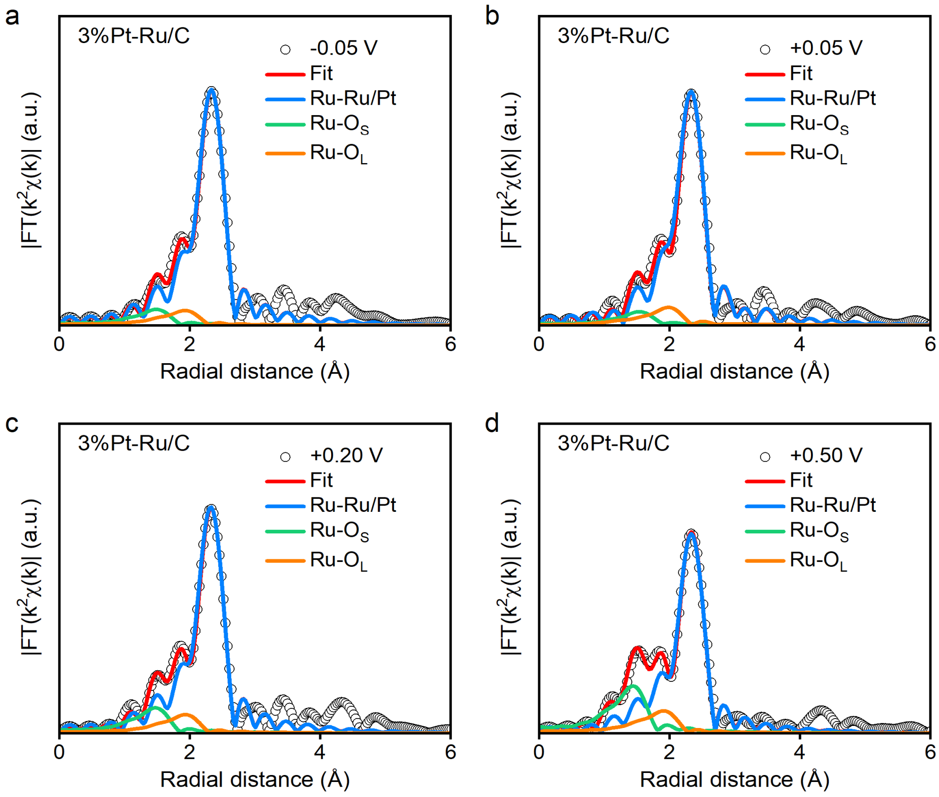

Supplementary Figure 28. Ru-K edge EXAFS fitting of 3%Pt-Ru/C with Ru-O$_S$, Ru-O$_L$ and Ru-Ru paths at -0.05 V (**a**), +0.05 V (**b**), +0.20 V (**c**) and +0.50 V (**d**).

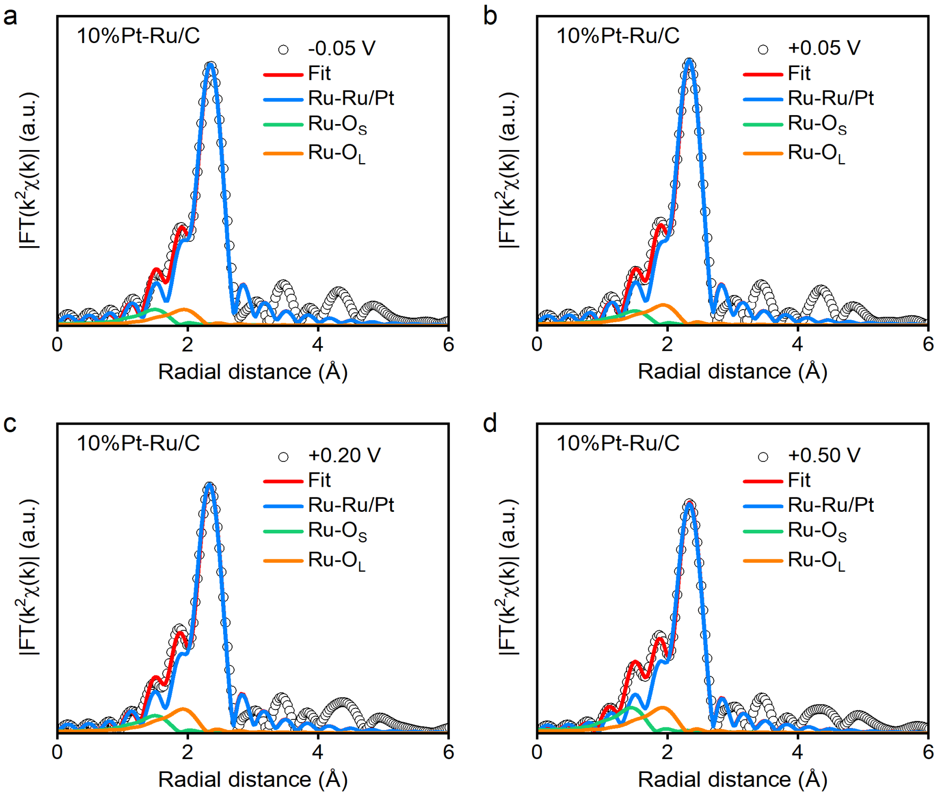

Supplementary Figure 29. Ru-K edge EXAFS fitting of 10%Pt-Ru/C with Ru-O_S, Ru-O_L and Ru-Ru paths at -0.05 V (**a**), +0.05 V (**b**), +0.20 V (**c**) and +0.50 V (**d**).

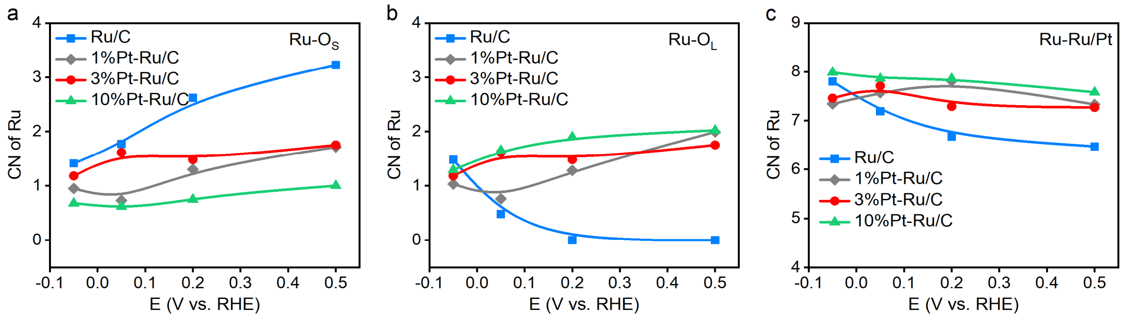

Supplementary Figure 30. Coordination numbers (CN) of Ru-O_S (**a**), Ru-O_L (**b**) and Ru-Ru/Pt (**c**) paths obtained from Ru-K edge EXAFS fitting results of Ru/C and Pt-Ru/C with different Pt loading. Ru/C and Pt-Ru/C samples show similar coordination structure at low potential (-0.05 V). As potential increases, Ru/C undergoes severe oxidation, showing increased Ru-O_S bond and decreased Ru-Ru and Ru-O_L bond. For Pt-Ru/C with different Pt loading, material oxidation is suppressed and the essential Ru-O_L is preserved.

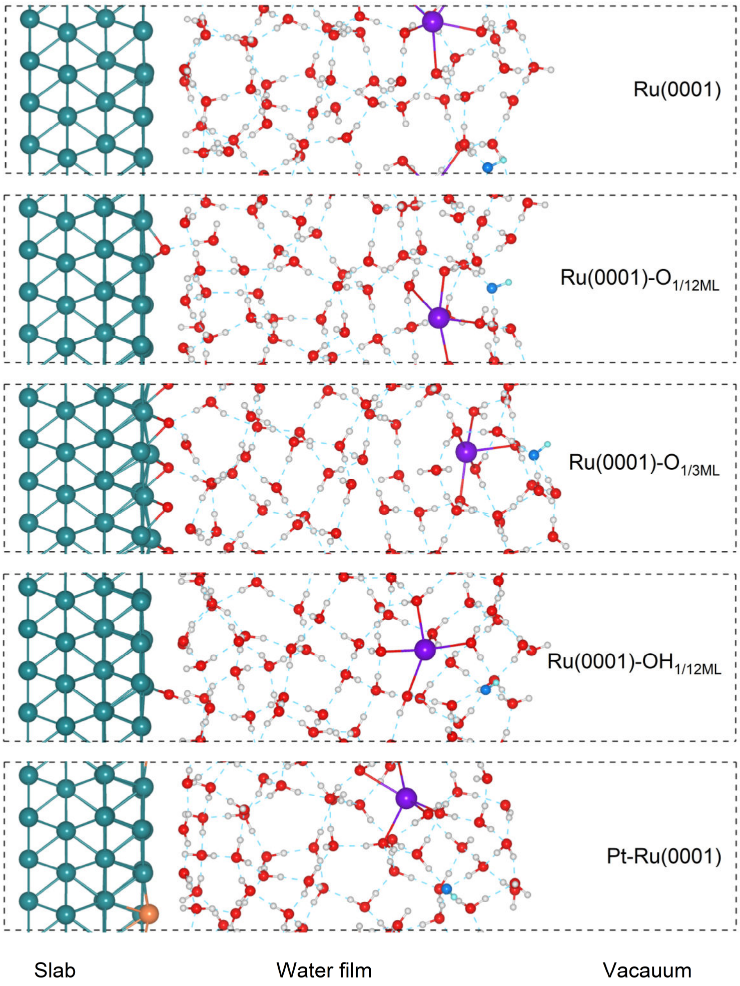

Supplementary Figure 31. Representative snapshots of the interfacial structure on the Ru(0001), Ru(0001)-$O_{1/12ML}$, Ru(0001)-$OH_{1/12ML}$, Ru(0001)-$O_{1/3ML}$ and Pt-Ru(0001) surface at 15 ps.

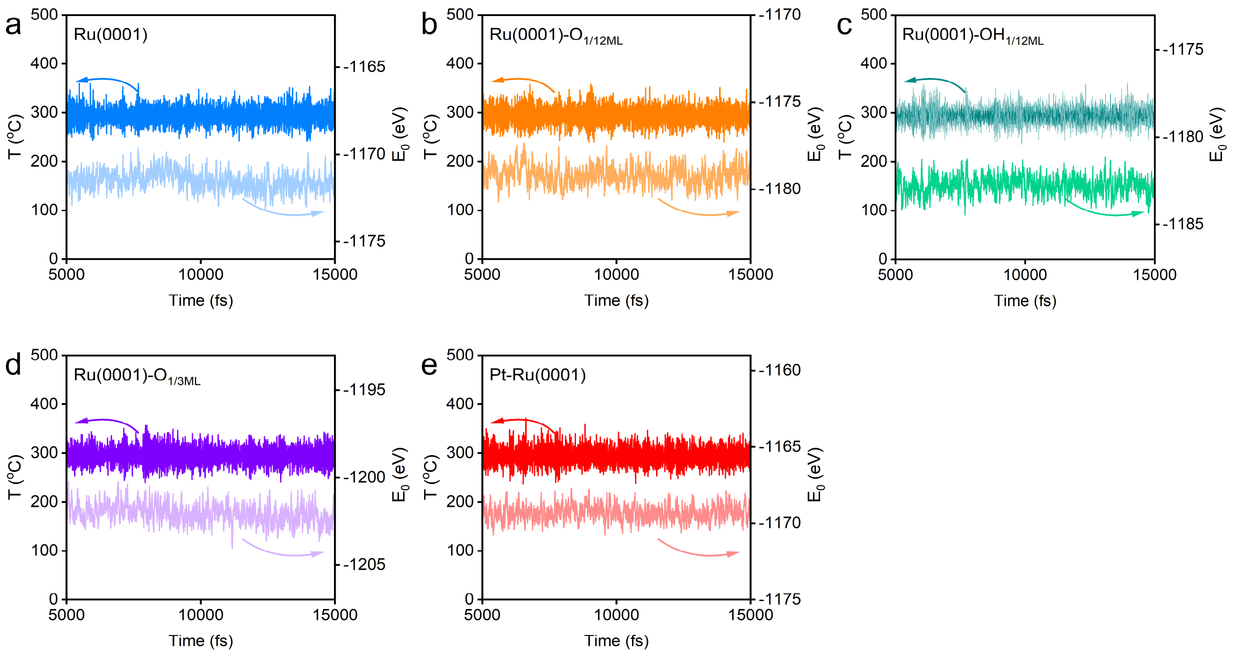

Supplementary Figure 32. A selected paradigm of temperature and potential energy evolution during AIMD simulations of Ru(0001)/H_2O (**a**), Ru(0001)-$O_{1/12ML}$/H_2O (**b**), Ru(0001)-$OH_{1/12ML}$/H_2O (**c**), Ru(0001)-$O_{1/3ML}$/H_2O (**d**) and Pt-Ru(0001)/H_2O (**e**) interfaces at 300 K. The constant temperature and energy during the simulation verify the stable structure during the AIMD simulation.

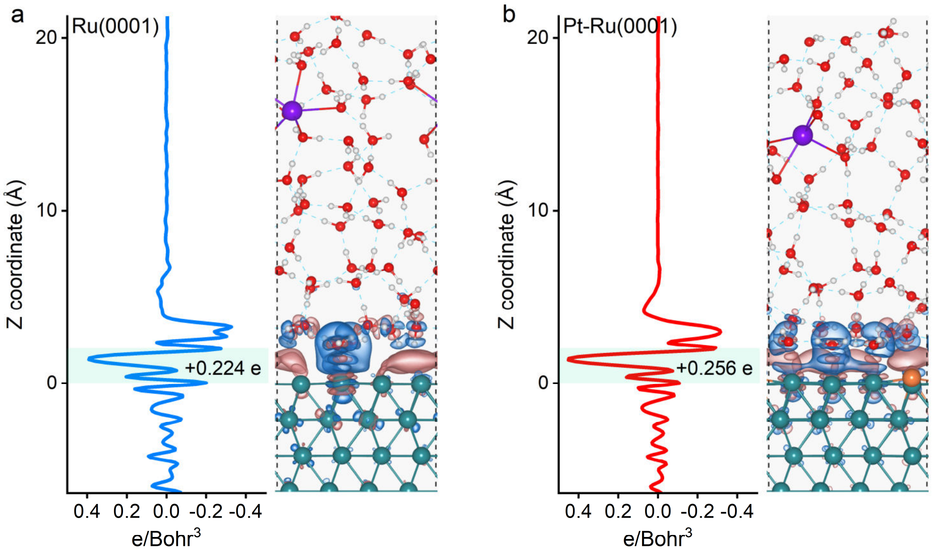

Supplementary Figure 33. Charge density difference maps of the Ru(0001)/H_2O interface (**a**) and Pt-Ru(0001)/H_2O interface (**b**). (Isosuface value: 0.002 e $Å^{-3}$; blue: charge consumption; red: charge accumulation).

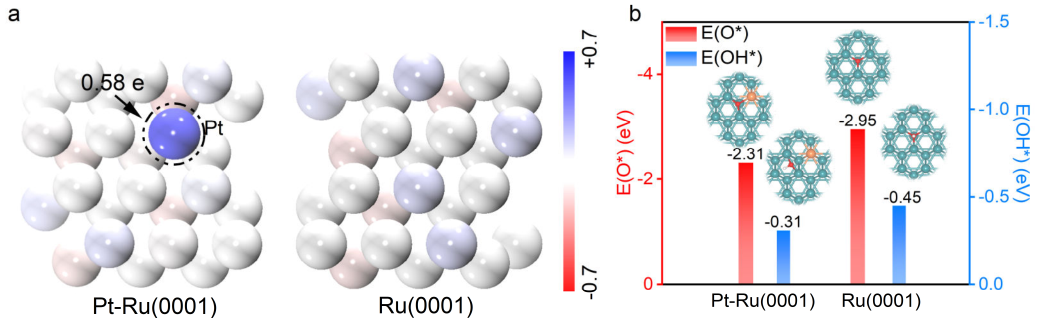

Supplementary Figure 34. a, Bader charge analysis of Ru(0001) and Pt-Ru(0001) surfaces. **b,** The adsorption energies of O* and OH* on Ru(0001) and Pt-Ru(0001) surfaces. Compared with small charge transfer on pure Ru surface, the incorporation of Pt alters the electron density of the surrounding Ru atoms and leads to electron transfer from Ru to Pt (0.58 e), indicating enriched electron density around Pt site. Furthermore, the electron-enriched sites possess weakened adsorption of OH* and O* compared with pure Ru surface.

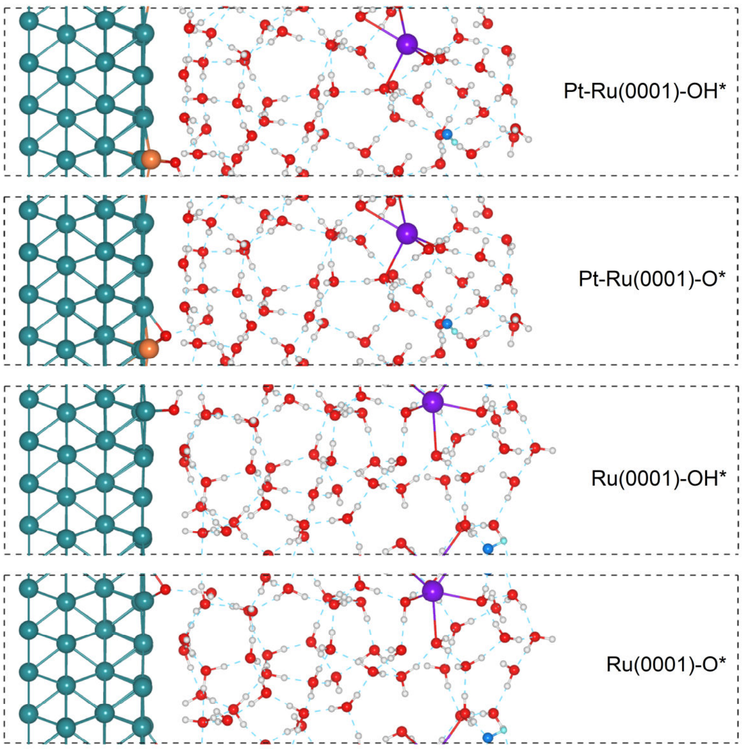

Supplementary Figure 35. Adsorption configurations of O* and OH* on Ru(0001)/H_2O (labeled as Ru(0001)-O* and Ru(0001)-OH*) and Pt-Ru(0001)/H_2O (labeled as Pt-Ru(0001)-O* and Pt-Ru(0001)-OH*).

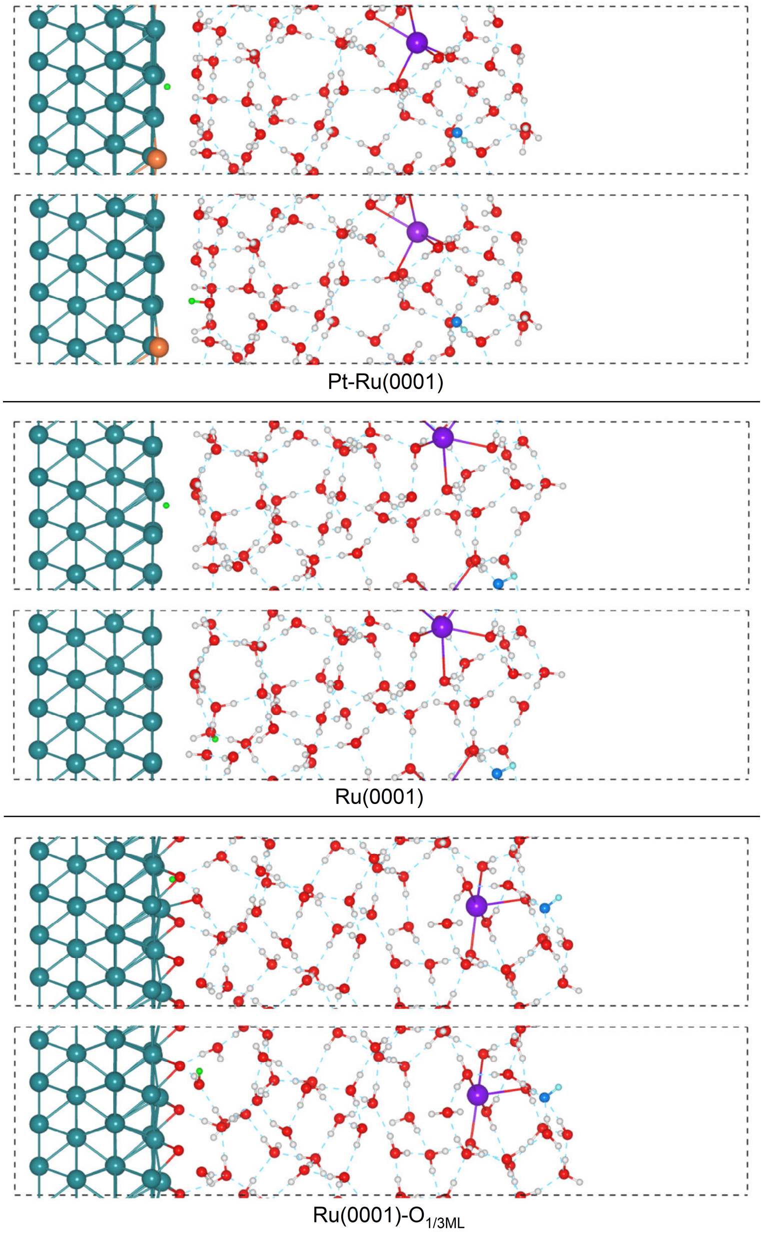

Supplementary Figure 36. Enlarged structural information during a surface-adsorbed H* releasing to interfacial water layer on Pt-Ru(0001), Ru(0001) and Ru(0001)-$O_{1/3ML}$ for the interfacial structures obtained at 15 ps.

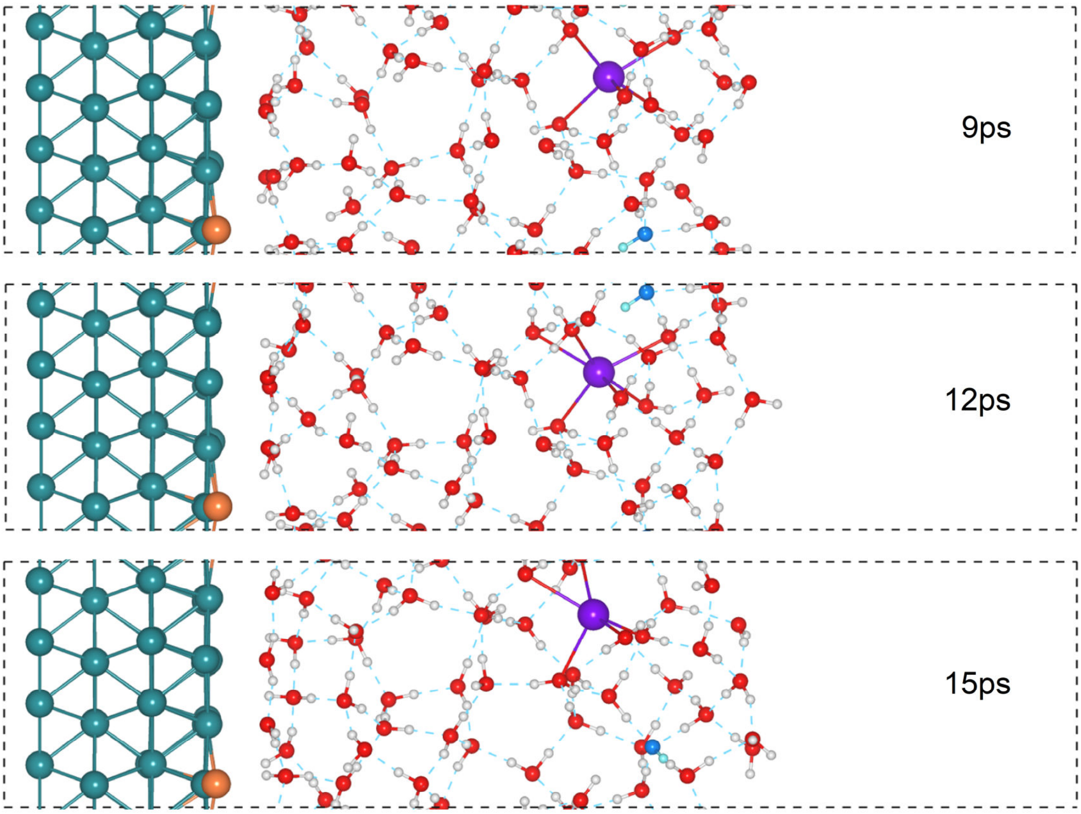

Supplementary Figure 37. Snapshots of the interfacial structure on the Pt-Ru(0001) surface at 9, 12 and 15 ps, respectively, which display different water structure during the simulation.

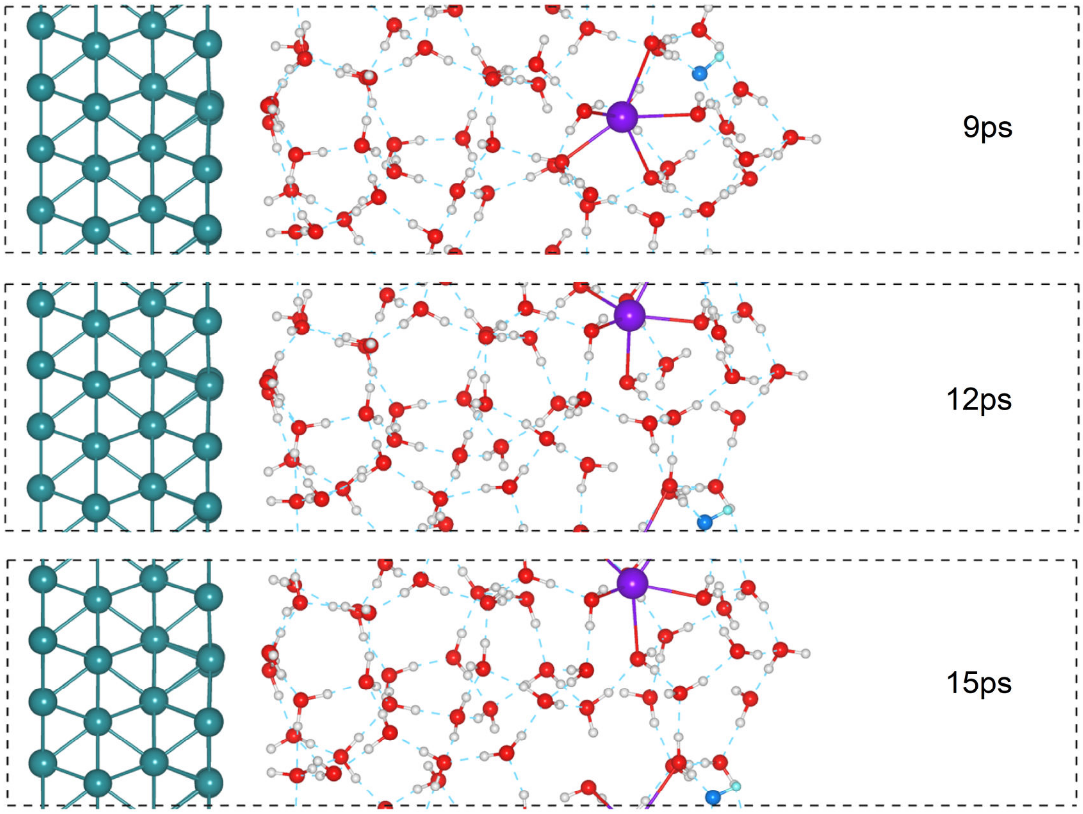

Supplementary Figure 38. Snapshots of the interfacial structure on the Ru(0001) surface at 9, 12 and 15 ps, respectively, which display different water structure during the simulation.

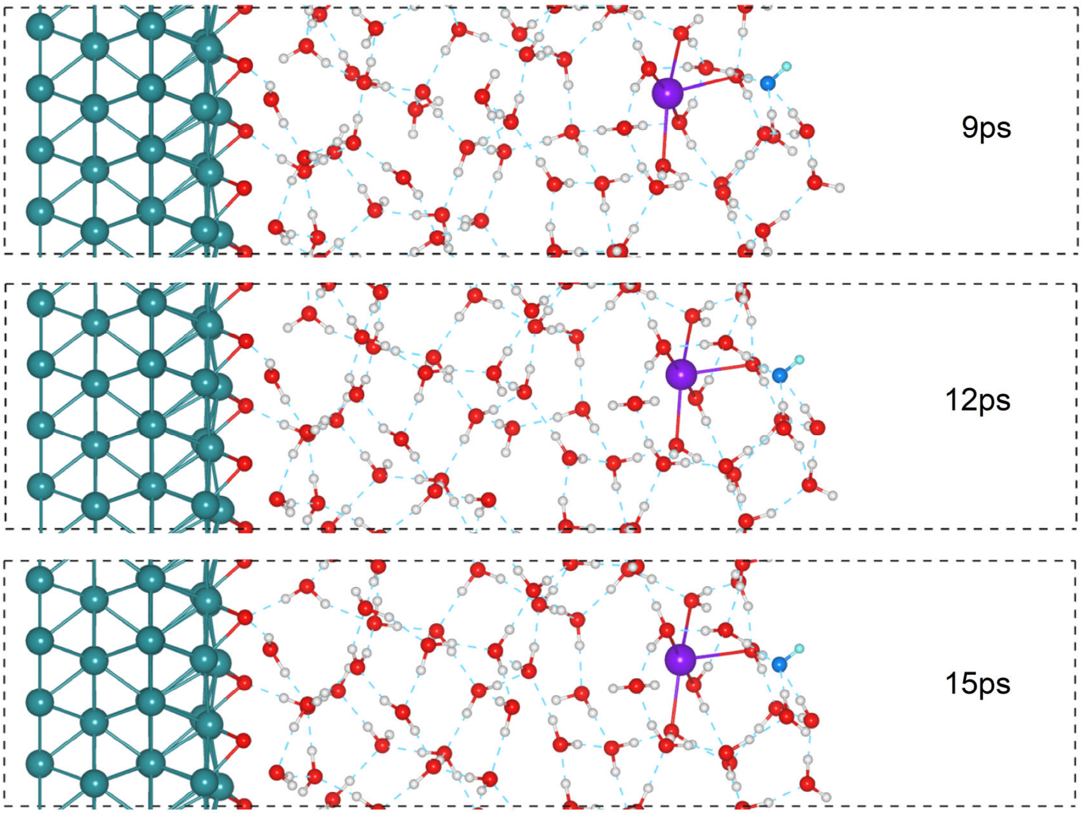

Supplementary Figure 39. Snapshots of the interfacial structure on the Ru(0001)-$O_{1/3ML}$ surface at 9, 12 and 15 ps, respectively, which display different water structure during the simulation.

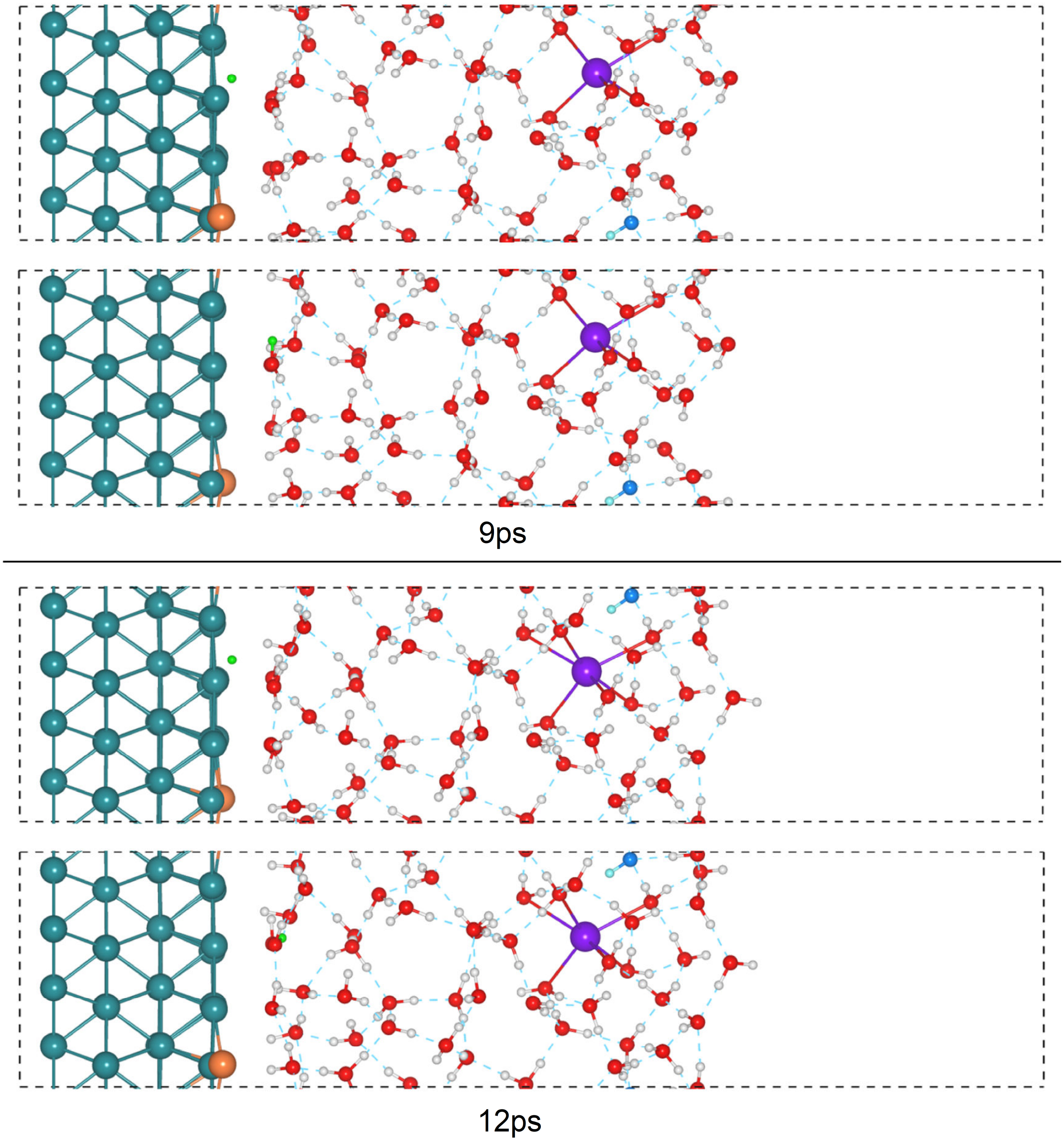

Supplementary Figure 40. Enlarged structural information during a surface-adsorbed H* releasing to interfacial water layer on Pt-Ru(0001) for the interfacial structures obtained at 9 and 12 ps.

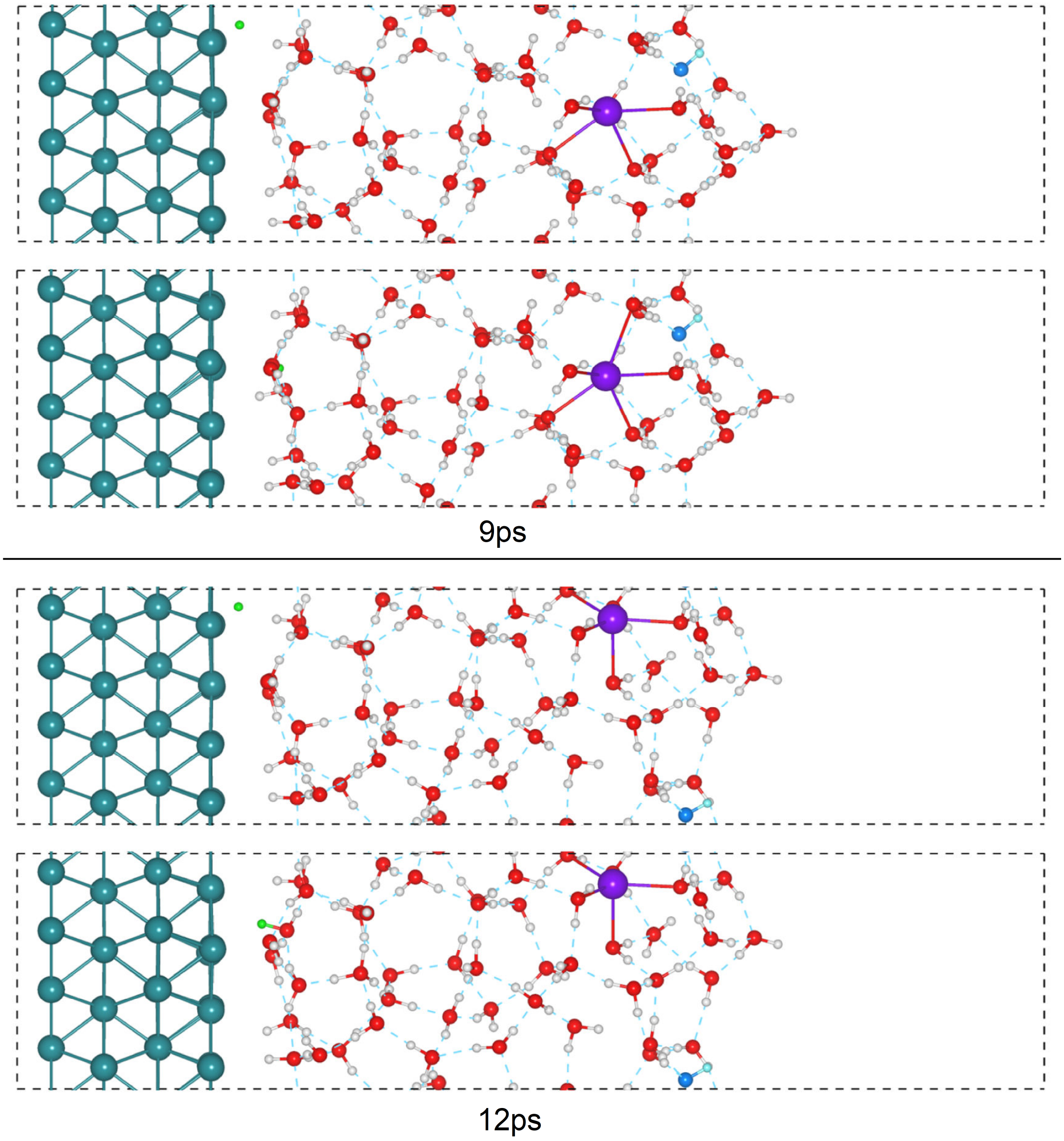

Supplementary Figure 41. Enlarged structural information during a surface-adsorbed H* releasing to interfacial water layer on Ru(0001) for the interfacial structures obtained at 9 and 12 ps.

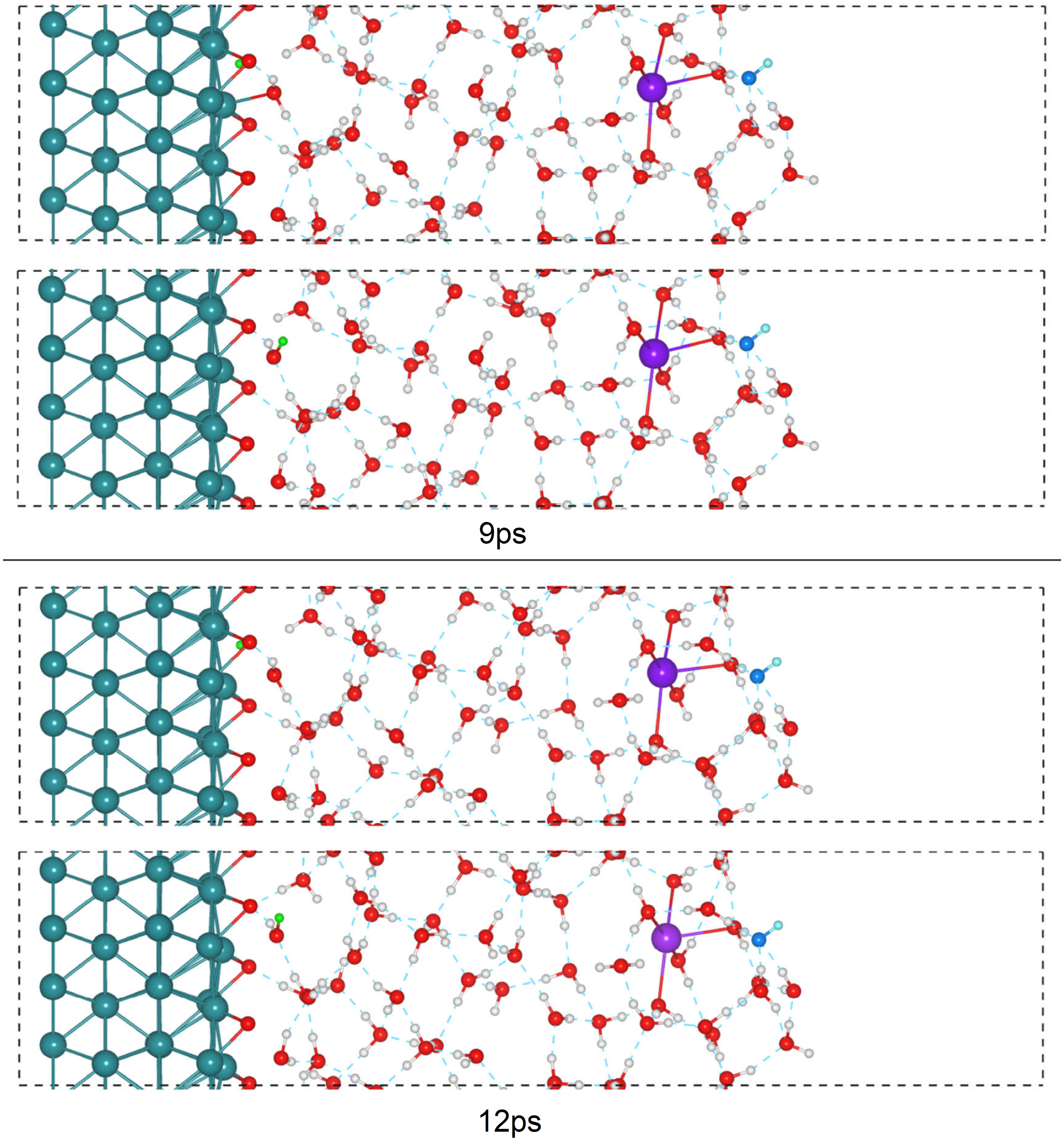

Supplementary Figure 42. Enlarged structural information during a surface-adsorbed H* releasing to interfacial water layer on Ru(0001)-$O_{1/3ML}$ for the interfacial structures obtained at 9 and 12 ps.

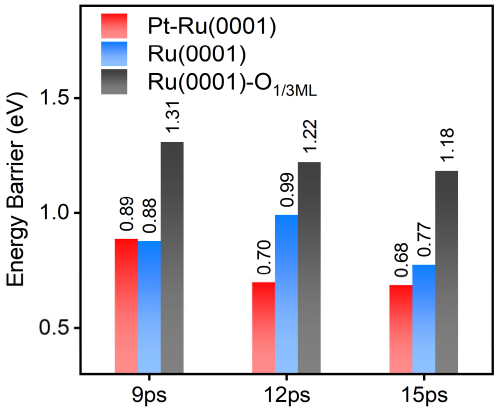

Supplementary Figure 43. The thermodynamic energy barriers for H* release from surface to electrolyte based on the water structure obtained at 9, 12 and 15ps. To understand whether the choice of exact water structure/water network has a huge effect on the H* desorption thermodynamics, the different structures at 9 ps and 12 ps during the AIMD simulations were obtained. The optimized structures are presented in Supplementary Fig. 36-38, which show different water network structures. Based on the thermodynamic results, the different water structures from different snapshots will lead to different H* releasing thermodynamic energy barrier. However, the total trend remains the same that the introduction of Pt would facilitate H* desorption while the oxidized Ru(0001)-$O_{1/3ML}$ surface always possesses hindered H* desorption.

Supplementary Table 1. Pt-L_3 edge EXAFS fitting parameters of Pt/C at different potentials. (S_0^2 = 0.794)

Potential	Path	R (Å)	CN	σ^2	E_0	R_f%
+0.05 V	Pt-O_L	/	/	/	8.4	0.2
	Pt-Pt	2.76	10.1	0.004		
+0.20 V	Pt-O_L	/	/	/	8.0	0.5
	Pt-Pt	2.76	10.6	0.004		
+0.50 V	Pt-O_L	/	/	/	8.3	0.3
	Pt-Pt	2.76	10.1	0.004		

Supplementary Table 2. Pt-L_3 edge EXAFS fitting parameters of 1%Pt-Ru/C at different potentials. (S_0^2 = 0.794)

Potential	Path	R (Å)	CN	σ^2	E_0	R_f%
+0.05 V	Pt-O_L	2.52	3.2	0.004	-0.17	2.0
	Pt-Ru	2.66	5.9	0.006		
+0.20 V	Pt-O_L	2.55	2.3	0.003	2.27	1.4
	Pt-Ru	2.67	6.3	0.006		
+0.50 V	Pt-O_L	2.54	2.4	0.006	2.03	0.9
	Pt-Ru	2.68	6.2	0.007		

Supplementary Table 3. Pt-L_3 edge EXAFS fitting parameters of 3%Pt-Ru/C at different potentials. (S_0^2 = 0.794)

Potential	Path	R (Å)	CN	σ^2	E_0	R_f%
+0.05 V	Pt-O_L	2.53	3.2	0.003	2.80	0.7
	Pt-Ru	2.69	5.7	0.010		
+0.20 V	Pt-O_L	2.53	3.3	0.003	0.03	1.2
	Pt-Ru	2.67	6.3	0.010		
+0.50 V	Pt-O_L	2.53	3.2	0.002	-0.76	0.8
	Pt-Ru	2.67	6.0	0.010		

Supplementary Table 4. Ru-K edge EXAFS fitting parameters of Ru/C at different potentials. (S_0^2 = 0.687)

Potential	Path	R (Å)	CN	σ^2	E_0	R_f%
-0.05 V	Ru-O_S	2.05	1.4	0.005	-4.84	1.4
	Ru-O_L	2.45	1.5	0.005		
	Ru-Ru	2.65	7.8	0.005		
+0.05 V	Ru-O_S	2.02	1.8	0.005	-5.14	1.2
	Ru-O_L	2.56	0.5	0.005		
	Ru-Ru/Pt	2.64	7.2	0.006		
+0.20 V	Ru-O_S	2.03	2.6	0.005	-2.11	0.9
	Ru-O_L	/	/	/		
	Ru-Ru/Pt	2.65	6.7	0.006		
+0.50 V	Ru-O_S	2.01	3.2	0.005	-2.48	1.3
	Ru-O_L	/	/	/		
	Ru-Ru/Pt	2.66	6.5	0.007		

Supplementary Table 5. Ru-K edge EXAFS fitting parameters of 1%Pt-Ru/C at different potentials. (S_0^2 = 0.687)

Potential	Path	R (Å)	CN	σ^2	E_0	R_f%
	Ru-O_S	2.02	1.0	0.005	-5.34	0.7
-0.05 V	Ru-O_L	2.52	1.0	0.005		
	Ru-Ru	2.64	7.3	0.005		
+0.05 V	Ru-O_S	2.01	0.7	0.005	-5.39	1.0
	Ru-O_L	2.54	0.8	0.005		
	Ru-Ru/Pt	2.64	7.6	0.005		
+0.20 V	Ru-O_S	2.02	1.3	0.005	-5.45	1.0
	Ru-O_L	2.5	1.3	0.005		
	Ru-Ru/Pt	2.64	7.8	0.006		
+0.50 V	Ru-O_S	1.99	1.7	0.005	-6.05	0.9
	Ru-O_L	2.50	2.0	0.005		
	Ru-Ru/Pt	2.64	7.3	0.006		

Supplementary Table 6. Ru-K edge EXAFS fitting parameters of 3%Pt-Ru/C at different potentials. (S_0^2 = 0.687)

Potential	Path	R (Å)	CN	σ^2	E_0	R_f%
-0.05 V	Ru-O_S	2.04	0.7	0.005	-5.68	0.8
	Ru-O_L	2.52	1.2	0.005		
	Ru-Ru	2.64	7.5	0.005		
+0.05 V	Ru-O_S	2.05	0.5	0.005	0.55	1.0
	Ru-O_L	2.58	1.6	0.005		
	Ru-Ru/Pt	2.64	7.7	0.005		
+0.20 V	Ru-O_S	2.02	1.0	0.005	-5.72	0.6
	Ru-O_L	2.52	1.5	0.005		
	Ru-Ru/Pt	2.63	7.3	0.005		
+0.50 V	Ru-O_S	1.98	1.8	0.005	-6.06	0.8
	Ru-O_L	2.51	1.8	0.005		
	Ru-Ru/Pt	2.65	7.3	0.006		

Supplementary Table 7. Ru-K edge EXAFS fitting parameters of 10%Pt-Ru/C at different potentials. (S_0^2 = 0.687)

Potential	Path	R (Å)	CN	σ^2	E_0	R_f%
-0.05 V	Ru-O_S	2.04	0.7	0.005	-4.85	1.0
	Ru-O_L	2.53	1.3	0.005		
	Ru-Ru	2.65	7.99	0.005		
+0.05 V	Ru-O_S	2.05	0.6	0.005	-5.86	0.7
	Ru-O_L	2.53	1.7	0.005		
	Ru-Ru/Pt	2.65	7.8	0.005		
+0.20 V	Ru-O_S	2.05	0.8	0.005	-5.45	0.9
	Ru-O_L	2.52	1.9	0.005		
	Ru-Ru/Pt	2.64	7.9	0.005		
+0.50 V	Ru-O_S	1.99	1.0	0.005	-5.79	0.4
	Ru-O_L	2.52	2.0	0.005		
	Ru-Ru/Pt	2.65	7.6	0.005		
